# A Network Analysis of Major Depressive Disorder Symptoms and Age- and Gender-Related Differences in People over 65 in a Madrid Community Sample (Spain)

**DOI:** 10.3390/ijerph17238934

**Published:** 2020-12-01

**Authors:** Miguel Ángel Castellanos, Berta Ausín, Sara Bestea, Clara González-Sanguino, Manuel Muñoz

**Affiliations:** 1 Methodology in Behavioral Sciences Department, Campus de Somosaguas, School of Psychology, Psychobiology, Complutense University of Madrid, Ctra. de Húmera, s/n, 28223 Pozuelo de Alarcón, Madrid, Spain; mcastellanos@psi.ucm.es (M.Á.C.); sbestea@ucm.es (S.B.); 2 Evaluation and Clinical Psychology Department, Campus de Somosaguas, School of Psychology, Personality, Complutense University of Madrid, Ctra. de Húmera, s/n, 28223 Pozuelo de Alarcón, Madrid, Spain; clagon06@ucm.es (C.G.-S.); mmunozlo@ucm.es (M.M.)

**Keywords:** major depressive disorder, elderly people, gender differences, network analysis

## Abstract

Major depressive disorder (MDD) is one of the most prevalent conditions among mental disorders in individuals over 65 years. People over 65 who suffer from MDD are often functionally impaired, chronically physically ill, and express cognitive problems. The concordance between a clinician-assessed MDD diagnosis in a primary care setting and MDD assessed with a structured clinical interview in older adults is only approximately 18%. Network analysis may provide an alternative statistical technique to better understand MDD in this population by a dimensional approach to symptomatology. The aim of this study was to carry out a network analysis of major depressive disorder (MDD) in people over 65 years old. A symptom network analysis was conducted according to age and gender in 555 people over 65, using a sample from the MentDis_ICF65+ Study. The results revealed different networks for men and women, and for the age groups 65–74 and 75–84. While depressive mood stood out in women, in men the network was more dispersed with fatigue or loss of energy and sleep disturbances as the main symptoms. In the 65–74 age group, the network was complex; however, in the 75–84 age group, the network was simpler with sleep disturbances as the central symptom. The gaps between the networks indicate the different characteristics of MDD in the elderly, with variations by gender and age, supporting the idea that MDD is a complex dynamic system that has unique characteristics in each person, rather than a prototypical classification with an underlying mental disorder. These unique characteristics can be taken into account in the clinical practice for detection and intervention of MDD.

## 1. Introduction

Major depressive disorder (MDD) is considered as one of the major problems related to mental health nowadays given its significant impact on the quality of life and functionality of the affected people, as well as its high prevalence rates. The most recent European epidemiological study on the mental health of people over 65 states that 11.6% suffered from MDD over the last year, with women and people between 65 and 74 years of age being the most affected [1]. In the Community of Madrid (Spain), 10.3% of people over 65 years had suffered from MDD in the last year [2]. However, MDD has an annual prevalence of 3.9% in the general population [3], which gives an idea of the magnitude of the problem in people over 65. People over 65 who suffer from MDD are often functionally impaired, chronically physically ill, and express cognitive problems. Furthermore, Andreas et al. [4] point out that the concordance between a clinician-assessed MDD diagnosis in a primary care setting and MDD assessed with a structured clinical interview in older adults is only approximately 18%. On the other hand, despite the high numbers of MDD in this population, which indicate the need to pay special attention to this problem, it is necessary to point out that in the current systems of diagnosis (Diagnostic and Statistical Manual of Mental Disorders (DSM-5); International Statistical Classification of Diseases and Related Health Problems (ICD-11)), no differences or specifications are found according to this age group, suggesting a series of common diagnostic criteria with the same importance, which perhaps in the elderly population may not represent the full extent of the disorder.

The classification of mental disorders may be done using either a dimensional or a categorical approach, depending on the purpose [5]. Both perspectives involve different approaches to the conceptualization of mental disorders, and a controversy has long existed about which of the two perspectives, categorical or dimensional, is more appropriate. Haslam [6] reviewed studies of the categorical versus dimensional status of mental disorders that employ taxometric methodology. This author found that categorical and dimensional models each receive well-replicated support for some groups of mental disorders. Studies favor categorical models for melancholia, eating disorders, pathological dissociation, and schizotypal and antisocial personality disorders. Dimensional models tend to be favored for the broad neurotic spectrum (general depression, generalized anxiety, posttraumatic stress disorder) and for borderline personality disorder.

For over a century, research in psychopathology has focused on categorical diagnostic classifications. Until a few years ago, the criteria specified in the diagnostic classifications in use (DSM-5 and ICD-11) guided mental health professionals in determining whether the problems affecting a person met all the criteria for a mental health disorder. Although such classifications have served to advance psychopathological assessment, the need for a dimensional approach to mental disorders is becoming increasingly evident. Traditional categorical approaches to psychiatric diagnosis emphasize hallmark symptoms that are strongly associated with a single disorder, but seldom associated with other disorders. These traditional conceptualizations of psychopathology presume that symptoms of mental disorders are reflective of underlying diseases. Importantly, this approach has the potential to obscure important differences between specific symptoms, as well as relationships between symptoms. The potential influence of symptoms on the development of other symptoms is not uniformly distributed [7].

In recent years, evidence has been provided for the clinical usefulness of several alternative diagnostic perspectives. In this sense, the perspective of symptom networks in psychopathology, proposed by Borsboom [8], questions whether diagnostic criteria within the same mental disorder are independent of each other. Variables that have always been considered as indicators of latent variables should be taken as autonomous causal variables in a network of dynamic systems. For example, the diagnostic criteria for MDD (DSM-5) [9] include symptoms such as insomnia, fatigue, and a decreased ability to concentrate. In empirical studies, a person’s scores for each of these symptoms are combined to give a total score, which serves as a measure of depression. This way of diagnosing ignores the presence of direct relationships between symptoms. For example, lack of sleep causes fatigue, and this fatigue leads to problems with concentration. This relationship between variables, which are not necessarily symptoms, is made in the case formulation in clinical practice. In this line, Maj [10] argues that we do need current diagnostic categories, but the act of diagnosis is only one step in the process leading to the key aims of the optimal formulation of the management plan and the prediction of outcomes. On the other hand, Borsboom [8] establishes a clear difference between an illness and a mental disorder. An illness presupposes a known etiology (meaning that the symptoms are the consequence of a common cause), while a mental disorder refers to a syndromic constellation of symptoms that remain empirically linked, in such a way that the symptoms are related to each other. In other words, the symptoms constitute the disorder and form an interrelated network, meaning that the symptoms, as noted, are not the consequence of an underlying mental disorder. The causal systems perspective [11] describes the possibility that symptom co-occurrence is due to direct symptom-to-symptom relationships rather than a common cause, in such a way that symptoms are constitutive of mental disorder, not reflective of it. Fried et al. [12] highlighted that the symptoms for MDD defined in the DSM-5 differ markedly from symptoms assessed in common rating scales, and the empirical question about core depression symptoms remains unresolved. These authors conclude that the network perspective supports neither the standard psychometric notion that depression symptoms are equivalent indicators of MDD, nor the common assumption that DSM symptoms of depression are of higher clinical relevance than non-DSM depression symptoms.

The possible number of combinations of MDD symptomatology has become evident in different studies [6,10,13,14,15,16,17]. Consequently, it is possible that individuals with a DSM-5 MDD diagnosis can have remarkably distinct symptom presentations. Diagnostic systems like the DSM-5 or ICD-11 can be considered to partly reflect the structure of psychopathology through patterns of symptom overlap. A straightforward way of studying such patterns is by representing individual symptoms as nodes in a network and connecting them whenever they feature as symptoms of the same disorder [17]. From this perspective, symptoms are not reflective of an underlying disorder; instead, the associations among symptoms constitute the disorder. Networks consist of nodes and edges. Nodes represent the objects of study, and edges represent the connections between them. In psychopathology networks, nodes represent symptoms and edges represent associations between symptoms [18], allowing the creation of two-dimensional maps formed by nodes, grouped together as they are related and indicating the strength of the connection with the thickness of the edges that connect them. In this way, it is possible to study the interrelationships of the symptoms from a visual perspective, facilitating the interpretation of the results and helping to understand the structure of underlying relationships, identifying which tend to appear together and connected and which are more peripheral.

In addition to this lack of representation of relationships, another peculiarity of the traditional diagnostic classifications is that they do not indicate differences in the diagnostic criteria by age and gender. As indicated, in the case of DMM, its prevalence has been found to be higher in older people than in other age groups, perhaps suggesting the presence of certain particularities of this group that make the presence of the diagnosis more frequent, and that therefore may also affect its characteristics. In addition, in relation to gender, from the Gender Response Framework, it has been pointed out how men tend to show typically more externalizing male traits, such as somatic symptomatology or anger, while in women it is more common to have internalized symptoms [19,20,21]. Thus, in relation to MDD it would be expected to find differences in symptomatology according to gender, which could facilitate more appropriate assessments that would favor early detection and a more accurate diagnosis of the problem.

To date, only a few studies have employed network analysis of MDD symptoms [6,14,16,17] and despite the demonstrated prevalence of this disorder in the elderly and the fact that MDD in older people shows differences in terms of etiological factors, clinical presentation, and outcomes of interventions, than MDD in people under 65, only one of these studies has conducted a network analysis focusing on MDD in older people [22]. In this study, they used a 12-item screening instrument to assess depressive symptoms (EURO-D) [23] These authors note that death wishes, depressed mood, loss of interest, and pessimism constitute the “backbone” that sustains depressive symptoms in late-life, and sex or age did not significantly influence the network structure. We hypothesize that, with the use of a structured diagnostic interview adapted to the symptomatology of MDD in people over 65, it is possible to construct different networks based on gender and age in the MDD. The current study is based on national data from the study Health and well-being of people between 65 and 84 years in Europe (MentDis_ICF65+ Study), which is a contemporary, nationally representative study of older adults. The objectives proposed are the following: (1) to construct MDD networks based on the DSM-IV-TR criterion symptoms; (2) to estimate the network structure among MDD symptoms and analyze age- and gender-related differences in a sample of people over 65 years old in the Community of Madrid (Spain).

## 2. Materials and Methods

### 2.1. Design

The sample was obtained within the framework of the MentDis_ICF65+ Study (Health and well-being of people between 65 and 84 years old in Europe) [24]. This longitudinal study was conducted in six European cities [24]. The sample was randomly selected from the population aged over 65 but younger than 84 in all districts of Madrid (Spain) and in a representative sample of other cities and rural areas of the Region of Madrid. It was stratified by age and gender.

Informed consent was requested from the people in the sample. The study was conducted in accordance with the Declaration of Helsinki, and the protocol was approved by the Deontological Commission of the Faculty of Psychology of the Complutense University of Madrid, with reference No. 2203201, and the European Commission.

### 2.2. Participants

A total of 555 people who met the inclusion criteria were interviewed.

The inclusion criteria were as follows: 1. Living in the Community of Madrid; 2. Between 65 and 84 years of age; 3. Able to provide informed consent to participate in the study. The exclusion criteria for the sample were as follows: 1. Presenting a severe cognitive impairment as evaluated using a Mini-Mental State Examination [25] cut-off point of >18; 2. Having a language barrier that prevented an interview taking place.

### 2.3. Variables and Instruments

The following variables and instruments were included in the assessment:

Sociodemographic variables: questions developed ad hoc allowed data collection on age, gender identity, marital status, educational level, economic situation (subjective perception from very bad to very good), importance of religious beliefs, and presence of medical diagnosis.

Evaluation of major depression disorder: To evaluate and diagnose major depression disorder, the Composite International Diagnostic Interview for people over 65 years (CIDI65+) [26] was applied. This standardized diagnostic interview was used to collect the lifetime, 12 month, and current prevalence data of mental disorders among elderly people. The interview evaluates the main disorders included in the DSM-IV-TR (Diagnostic and Statistical Manual of Mental Disorders—Revised). Thus, the CIDI65+ yields diagnosis based on the criteria of the DSM-IV-TR classification system [27]. The test–retest reliability of this interview is acceptable for major depression disorder (ⱪ = 0.55 [26]).

There are nine diagnostic criteria of DSM-5 [9] for major depression disorder, as in the DSM-IV-TR: depressed mood; loss of interest; appetite or weight disturbance; sleep difficulties; psychomotor agitation or retardation; fatigue or loss of energy; feelings of worthlessness; diminished ability to think or concentrate; suicidality. Neither the criteria nor the requirement for a duration of at least two weeks has changed, although the wording is not exact. In the DSM-IV-TR [27], no major depressive disorder was diagnosed if depressive symptoms existed for less than two months after the death of a loved one. This grief exclusion has been removed from DSM-5.

### 2.4. Analysis

The frequencies and percentages of the items in the CIDI65+ interview were calculated as descriptive statistics. A test for two proportions (Z-test) was calculated to compare the proportions of each symptom in each variable under study, gender (female, male) and age (group between 65–74 and 75–84 years).

R statistical software. (R Foundation for Statistical Computing, Vienna, Austria) The network was estimated using the InsingFit package created by van Borkulo [28]; this package implements a procedure called eLasso. This procedure is an extension of the lasso procedure that is widely used for continuous data and that imposes a l1-penalty on the estimation of the inverse covariance matrix. eLasso has been shown to work best when the data are binary without generating excessive computational overhead. The eLasso procedure is based on an adaptation to a binary space {0,1} of the Ising model that is widely used in physical sciences and that depends on two parameters, βjk (interaction between variables j and k) and τj (threshold of the variable to take the value 1). These parameters β and τ are estimated with logistic regressions in which for each variable both coefficients are calculated as a function of all the other variables in the network. These τ and β coefficients are finally used to represent the nodes and edges of the network. To guarantee the sparsity of the network, a l1-penalty is imposed on these estimated coefficients. This contraction is controlled by a penalty parameter that instead of being arbitrarily chosen, in this model is chosen based on the extended Bayesian information criterion (EBIC), which has been shown [1,28] to have good metric properties (converges with increasing sample size and has a low false-positive rate). The visualization of networks was carried out with the qgraph package [29] (gamma = 0.25). The centrality of each symptom is represented in a table with its raw values and in a graph with the standardized values for the statistics of strength, closeness, and betweenness, which are calculated as z-scores.

Estimates of the networks were made independently according to gender (male vs. female) and age (65–74 vs. 75–84). The estimation procedure was the same for all, but to facilitate the visual comparability of the results, the nodes of each group were forced to coincide positively using the layout found for the network with all subjects.

The stability of the network obtained for all subjects was estimated with the bootnet package by calculating the stability of the correlations between the edges against the loss of subjects using bootstrapping subsamples. The stability index CS (cor = 0.7) [30], used by Epskamp, reports the percentage of subsamples that have found a correlation between the original edge and those of the samples equal or higher than 0.7 and shows a reasonable value of stability equal or higher than 0.2.

The meanings of the acronyms used in the text, tables, and figures are as follows: DM—depressed mood; LI—loss of interest or pleasure; AW—appetite or weight disturbance; SD—sleep difficulties (insomnia or hypersomnia); PAR—psychomotor agitation or retardation; FE—fatigue or loss of energy; FW—feelings of worthlessness or excessive guilt; C—diminished ability to think or concentrate; SU—suicidality. The types of centrality analyses are as follows: node strength—sum of all the edges of one node with the others (estimate of how strongly it is connected to the rest); closeness—inverse of all the distances (number of edges crossed) shorter between one node and the others; betweenness—number of times a node appears on the shortest path between two other nodes.

The analyses have been performed using R (v3.5.6) with the nlme package [31].

## 3. Results

### 3.1. Characteristics of the Sample

The sample included 555 men and women between 65 and 84 years of age, with a mean age of 73.5 years. Table 1 shows the sample’s sociodemographic characteristics.

### 3.2. Symptomatology and Differences in Age and Gender

Descriptive statistics with frequencies and percentages of occurrence of each of the symptoms of CIDI65+ for the gender (female, male) and age groups (65–74, 75–84 years) are found in Table 2. The gender variable presents a greater presence of symptoms in women than in men, with these differences being significant for all symptoms except for psychomotor agitation. The greatest differences, expressed as the Chi-squared value of the Z-test, are given for DM (45% vs. 20%, X2 = 36.73), FE (34% vs. 13%, X2 = 32.14), and SD (36% vs. 16%, X2 = 27.46). In terms of the age variable, although there is a higher frequency of symptoms in the younger age group (65–7), statistically significant differences are only found for AW (21% vs. 13%, X2 = 5.99), PAR (16% vs. 8%, X2 = 5.74), and FW (21% vs. 14%, X2 = 4.79).

### 3.3. Gender-Based Networks

To facilitate interpretation of the results, a single network with all subjects was estimated and its layout was used as the layout for all the calculated subgroup networks. The stability statistics were calculated in this network.

**Figure 1:**
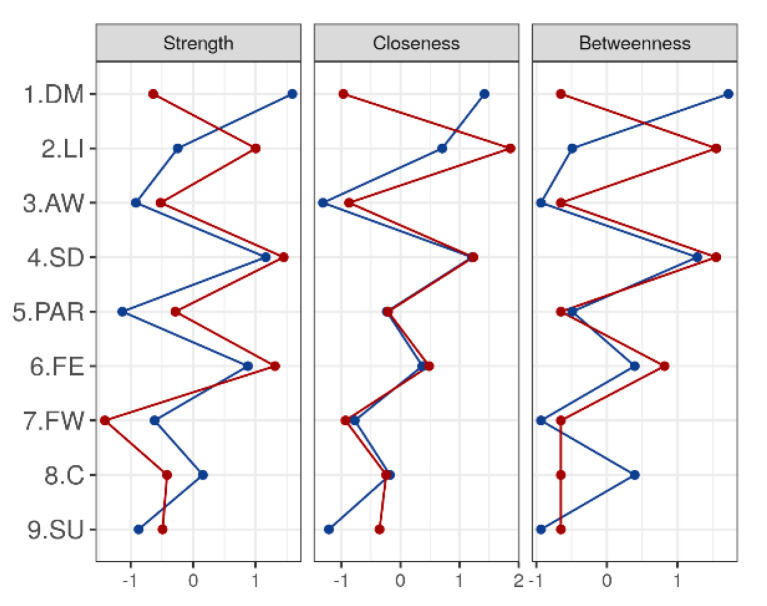
Centrality measures for CIDI65+ items by gender groups. DM: depressed mood; LI: loss of interest or pleasure; AW: appetite or weight disturbance; SD: sleep difficulties (insomnia or hypersomnia); PAR: psychomotor agitation or retardation; FE: fatigue or loss of energy; FW: feelings of worthlessness or guilt; C: diminished ability to think or concentrate; SU: suicidality; measures of centrality on CIDI65+ for women and men. The figure shows a plot of standardized values. Women are represented by the red line, and men by the blue line.

**Figure 2:**
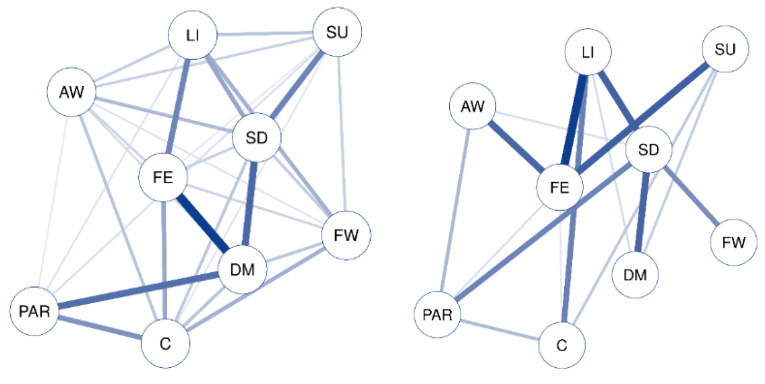
CIDI65+ symptoms network for females and males. Each node represents an item, and each link represents a relation between each pair of items (bolder lines indicate stronger relations). DM: depressed mood; LI: loss of interest or pleasure; AW: appetite or weight disturbance; SD: sleep difficulties (insomnia or hypersomnia); PAR: psychomotor agitation or retardation; FE: fatigue or loss of energy; FW: feelings of worthlessness or guilt; C: diminished ability to think or concentrate; SU: suicidality.

The networks for the symptoms measured by the CIDI65+ interview for women (left) and men (right) are found in Figure 1 and Figure 2, and the estimated values of centrality (strength, closeness, and betweenness) are found in Table 3.

Analyzing the structure of both networks, clear differences can be seen. The network for women is less sparse than that for men, with more non-zero edges between the nodes. The most central symptom is DM, which is strongly related to FE, and next strongly related to PAR and SD. The rest of the relationships are less intense, blurring the relationship of SD with SU, LI with FE, and PAR with C.

On the other hand, the men’s network structure is more sparse and more centered, with higher strength values in the relevant nodes and with fewer, but more intense, connections. Two differentiated structures can be seen, one formed by SU, LI, and AW, which are strongly related to FE (but not related to each other), and a second structure in which PAR, DM, and FW are related to SD (but not related to each other).

That is, DM is the symptom that plays a more central role in relationships for women, while for men this role is played by FE and SD, forming two sub-networks.

### 3.4. Age-Based Networks

**Figure 3:**
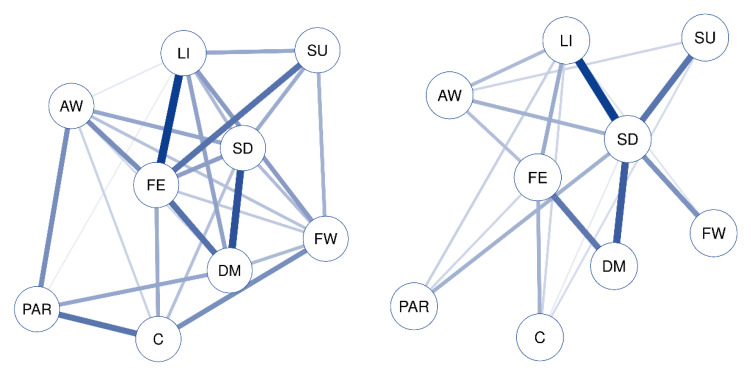
CIDI65+ symptoms network for age groups (age group 65–74 and age group 75–84). Each node represents an item, and each link represents a relation between each pair of items (bolder lines indicate stronger relations). DM: depressed mood; LI: loss of interest or pleasure; AW: appetite or weight disturbance; SD: sleep difficulties (insomnia or hypersomnia); PAR: psychomotor agitation or retardation; FE: fatigue or loss of energy; FW: feelings of worthlessness or guilt; C: diminished ability to think or concentrate; SU: suicidality.

**Figure 4:**
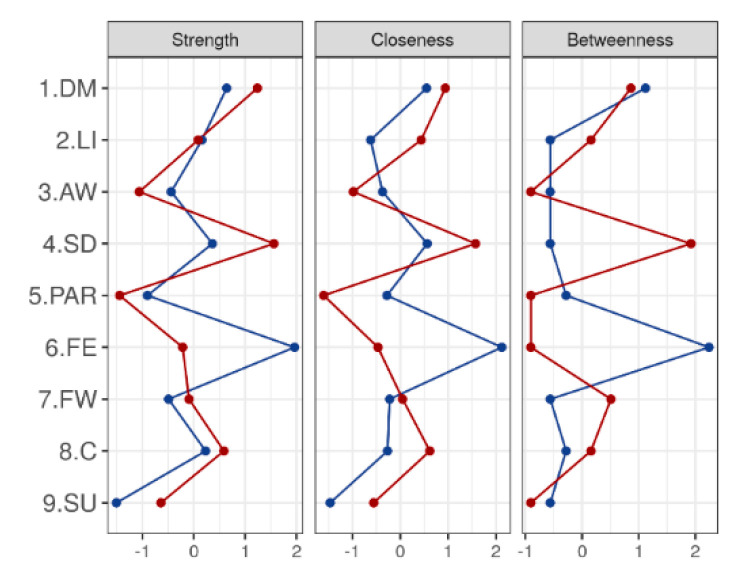
Centrality measures for CIDI65+ items by age groups. DM: depressed mood; LI: loss of interest or pleasure; AW: appetite or weight disturbance; SD: sleep difficulties (insomnia or hypersomnia); PAR: psychomotor agitation or retardation; FE: fatigue or loss of energy; FW: feelings of worthlessness or guilt; C: diminished ability to think or concentrate; SU: suicidality; measures of centrality on CIDI65+ for age groups. The figure shows a plot of standardized values. The 65–74 age group is represented by the blue line, and the 75–84 group by the red line.

The networks for the age group 65–74 (left) and the age group 75–84 (right) are shown in Figure 3 and Figure 4, and the table with central results is shown in Table 4.

The visual analysis of the network indicates that the group aged 65–74 presents a complex structure with many nodes and strong relationships between them, with FE being the symptom with the most centrality, as it maintains a strong relationship with LI, SU, and DM. The structure of the network for the group aged 75–84 is different, much sparser and with less intense relations between the nodes. A sub-network stands out, with SD as the central node (strength = 12.59), which is related to LI, SU, FW, and DM.

The value of network stability calculated with all subjects gave a CS (cor = 0.7) stability index of 0.360 for the edges, 0.205 for the intercept, and 0.517 for the strength values, which is within the reasonable values (greater than 0.2 for reasonable and greater than 0.5 for excellent) proposed by Epskamp, Borsboom, and Fried in 2018 [30].

## 4. Discussion

This is the first study in the scientific literature to use a standardized and structured clinical interview for mental disorders adapted to the needs of the elderly to report the network analysis of DSM-IV-TR MDD symptoms in the older adult population, analyzing gender and age differences. Our initial hypothesis regarding the possibility of developing a different network analysis according to gender and age of the MDD in elderly people is confirmed. The results reveal differences in the strength, closeness, and betweenness of the different networks according to gender and age, thus supporting the idea of MDD as a complex dynamic system [32] with unique characteristics in each person and not as a prototypical classification with an underlying mental disorder.

In relation to the networks developed in terms of gender, the main difference between men and women over 65 with MDD seems to be the presence of DM, which has a fundamental role in the case of women. However, in the case of men, DM has a more secondary role, emphasizing the loss of energy and problems of a physiological type, like alterations in sleep. The difference in networks based on gender in MDD was not found in a recent study conducted with adolescents [15], where the main differences between the two genders were only found in terms of body image and the relationship with self-hatred, which were more pronounced in teenage girls. Perhaps differences in MDD between males and females develop with exposure to life events and experiences, being greater in adulthood when an individual’s full development has already occurred, and differences in experiences may be more determined by gender. On the other hand, it has been shown that men and women have differences in the presentation of MDD symptoms. According the Gender Responding Framework previously commented on [19,20], Price et al. [21] confirmed that men who present and approve more typically male traits tend to present more externalizing-type symptoms. Our results seem to support this framework and findings, indicating that internalized symptomatology has a greater presence in women (DM), while in men externalizing symptoms are more central. These differences can be critical in making a more complete evaluation of the MDD as well as an early and more accurate diagnosis, being information that is not provided through traditional categorical classifications.

In relation to age, it is possible to observe changes in the model as age advances. In people aged 65–74, the network obtained is complex, with many nodes and relationships where FE and SD are the main symptoms. Nevertheless, the network seems to become simpler with the passage of time; in people aged 75–84. The change in network centrality over time is consistent with longitudinal studies, where it has been found that the centrality of a network can vary over time [33]. In this case, the change in the network stands out due to the disappearance of FE as a central symptom, which is no longer present in advanced ages, giving way to SD as the main symptom in MDD. Both networks can be key in designing interventions aimed at treating MDD in older people. While the emphasis is usually on the more emotional aspects of the disorder, the keys to addressing depression in older people may be in interventions that focus on behavioral activation or sleep enhancement.

Additionally, with regard to previous studies on MDD and networks, the review by Contreras et al. [34] indicates that DM and FE were found to be central symptoms and predictors of depression in several studies [35,36,37]. These results are consistent with those obtained in our study, where DM and FE are central symptoms in several networks. Moreover, it is worth mentioning that one of the symptoms of greatest weight in our results is SD, especially in men and in the older age group. These results have not been found in previous studies. However, the appearance of sleep alterations as a central symptom in MDD in elderly people and not in other age groups is consistent with several studies. These found that SD is frequent in advanced age [38,39], and seems to be especially related to depression in men [40,41]. Studies on MDD and networks have also highlighted the role of concentration as a central symptom [35,37], although in our results C was in no case central or strongly related to any other symptom. This lack of significance of concentration in our models is perhaps related to the exclusion criteria in the study of those people who presented cognitive impairment, a limitation that is pointed out below.

The results obtained in the different networks developed show that the symptomatology of MDD behaves differently in women and men, and also in different age groups in people over 65. As clinical repercussions, these differences seem to support the conceptualization of MDD from a dimensional point of view, proposing transdiagnostic models differentiated by gender and age, and closer, for example, to a Hierarchical Classification Model [42]. This type of model can be especially useful in evaluating the disorder. For a more effective detection of the disorder, more attention should be paid to the different ways of expressing the symptoms—internalizing or externalizing—as well as taking into account age when detecting symptoms. Other repercussions in clinical practice relate to intervention of the disorder, which perhaps should be approached using a clinical case formulation approach in which treatments are personalized, focusing on the symptoms that appear most frequently in each of the networks found, taking age and gender into account. Thus, in women it would presumably be more effective to carry out interventions at the emotional level, along with behavioral and sleep activation guidelines, while in men perhaps more useful interventions would focus on addressing somatic-type problems such as FE, and others such as LI, SD, AW, or SU.

One of the main limitations of the study is related to the representativeness of the sample. In the present study, exclusion criteria were applied for various technical reasons that were expected to make the evaluation process difficult: people with a severe cognitive deficit, those who were residents of a nursing home, homeless people, and non-Spanish speakers. It should also be noted that people over 85 were not included in the sample, which could have led to modifications in the developed network. People over 85 could perhaps be another group on which to develop further research on depressive symptomatology and develop a network of their own. On the other hand, the size of the sample and the sampling method do not allow us to generalize for the entire elderly population of the Community of Madrid, but they do offer a broad view of the situation. Moreover, excluding people with severe cognitive impairments is also a limitation when developing the MDD network. Excluding people with cognitive impairments from recruitment may have caused cases of pseudo-dementia to be omitted from the study, thus distorting the networks obtained. However, being aware of this limitation, it was decided that this criterion should be maintained because people with cognitive impairment cannot complete the CIDI65+. In addition, the results are based on the DSM-IV-TR criteria for MDD, and the use of the DSM-5 diagnoses could have led to different results. Since the basic characteristics have remained the same, one would not expect very different prevalence estimates.

## 5. Conclusions

From the perspective of network analysis, psychopathology is defended from a more flexible approach, giving greater weight to the centrality of the symptoms in a given disorder as well as the mutual dynamics between them [34]. The results obtained in the present study seem to support this perspective by showing different network analyses in people over 65 years old according to gender and age, which reveal the particular MDD characteristics for each group.

Network analysis has allowed a more in-depth observation of the characteristics of MDD in elderly people, a population group in which this disorder is often underdiagnosed. These results may be especially interesting for their clinical implications. They may help provide a more accurate diagnosis, predict the course of the disease [12], and lead to more effective interventions that address the most important, different, and characteristic symptoms and mutual relationships according to variables such as age and gender.

## Figures and Tables

**Table 1:** Sociodemographic characteristics of the sample (*N* = 555).

Socio-Demographic Characteristics	Nº	Prevalence (%)
**Gender**		
Male	267	48.1
Female	288	51.9
**Age (Average)**		
65–74	296	53.3
75–84	259	46.7
**Country born**		
Spain	547	98.6
Other	8	1.4
**Parents born in the same country**		
No	11	2
Yes	544	98
**Marital Status**		
Married	336	60.5
Separated	13	2.3
Divorced	28	5
Widower	151	27.2
Never been married	26	4.7
Other	1	0.2
Widower since (nº ages)	13.09 (0–50)	
**School/education**		
No	258	46.5
Yes	297	53.5
**Years of schooling**		
0–3	88	15.9
4–12	338	61.1
13+	127	23
**Work status**		
Retired	400	72.1
Homemaker/housewife	137	24.7
Working/employed	13	2.3
Unemployed	4	0.7
Other	1	0.2

**Table 2:** Frequencies and percentages of symptoms for gender and age variables in the Composite International Diagnostic Interview for people over 65 years (CIDI65+) interview (*N* = 555).

		Gender			Age Groups		
MDD Symptoms	Acronym	Female	Male	Z Test	Age Group 65–74	Age Group 75–84	Z-Test
ALL		288 (52%)	267 (48%)		292 (53%)	259 (47%)	
Depressed mood	DM	129 (45%)	54 (20%)	36.73 **	103 (35%)	80 (31%)	0.79
Loss of interest or pleasure	LI	77 (27%)	38 (14%)	12.44 **	68 (23%)	47 (18%)	1.68
Appetite or weight disturbance	AW	67 (23%)	28 (10%)	15.06 **	62 (21%)	33 (13%)	5.99 *
Sleep difficulties (insomnia or hypersomnia)	SD	104 (36%)	43 (16%)	27.46 **	84 (28%)	63 (24%)	0.97
Psychomotor agitation or retardation	PAR	40 (14%)	28 (10%)	1.19	46 (16%)	22 (8%)	5.74 *
Fatigue or loss of energy	FE	98 (34%)	35 (13%)	32.14 **	80 (27%)	53 (20%)	2.92
Feelings of worthlessness or excessive guilt	FW	68 (24%)	29 (11%)	14.74 **	62 (21%)	35 (14%)	4.79 *
Diminished ability to think or concentrate	C	65 (23%)	26 (10%)	15.72 **	54 (18%)	37 (14%)	1.3
Suicidality	SU	56 (19%)	20 (7%)	15.76 **	44 (15%)	32 (12%)	0.54

* *p* < 0.05. ** *p* < 0.001.

**Table 3:** Centrality measures for CIDI65+ items by gender groups.

	Strength		Closeness		Betweenness	
	Female	Male	Female	Male	Female	Male
DM	8.31	4.14	0.12	0.10	6	0
LI	5.66	8.76	0.11	0.17	1	15
AW	4.69	4.47	0.07	0.10	0	0
SD	7.69	10.01	0.12	0.16	5	15
PAR	4.38	5.14	0.09	0.12	1	0
FE	7.28	9.62	0.10	0.14	3	10
FW	5.12	1.97	0.08	0.10	0	0
C	6.24	4.76	0.09	0.12	3	0
SU	4.75	4.56	0.07	0.12	0	0

DM: depressed mood; LI: loss of interest or pleasure; AW: appetite or weight disturbance; SD: sleep difficulties (insomnia or hypersomnia); PAR: psychomotor agitation or retardation; FE: fatigue or loss of energy; FW: feelings of worthlessness or guilt; C: diminished ability to think or concentrate; SU: suicidality; measures of centrality on CIDI65+ for women and men. The table shows raw values.

**Table 4:** Centrality measures for CIDI65+ items by age groups.

	Strength		Closeness		Betweenness	
	65–74	75–84	65–74	75–84	65–74	75–84
DM	6.71	4.78	0.10	0.14	2	5
LI	6.50	7.50	0.11	0.14	1	0
AW	5.44	4.18	0.09	0.10	2	0
SD	7.15	12.59	0.11	0.16	0	17
PAR	4.01	2.75	0.08	0.09	2	0
FE	9.12	6.57	0.13	0.13	7	5
FW	6.07	2.23	0.10	0.10	0	0
C	4.94	2.93	0.09	0.08	1	0
SU	4.34	3.52	0.09	0.11	0	0

DM: depressed mood; LI: loss of interest or pleasure; AW: appetite or weight disturbance; SD: sleep difficulties (insomnia or hypersomnia); PAR: psychomotor agitation or retardation; FE: fatigue or loss of energy; FW: feelings of worthlessness or guilt; C: diminished ability to think or concentrate; SU: suicidality; measures of centrality on CIDI65+ for age groups. The table shows raw values.

## References

[1] Andreas S., Schulz H., Volkert J., Dehoust M., Sehner S., Suling A., Ausín B., Canuto A., Crawford M., Da Ronch C. (2017). Prevalence of mental disorders in elderly people: The European MentDis_ICF65+ study. Br. J. Psychiatry.

[2] Ausín B., Muñoz M., Castellanos M.A. (2017). Loneliness, Sociodemographic and Mental Health Variables in Spanish Adults over 65 Years Old. Span. J. Psychol..

[3] Haro J., Palacin C., Vilagut G., Martínez M., Bernal M., Luque I., Codony M., Dolz M., Alonso J. (2006). Prevalence of mental disorders and associated factors: Results from the ESEMeD-Spain study. Med. Clin..

[4] Andreas S., Dehoust M., Volkert J., Schulz H., Sehner S., Suling A., Wegscheider K., Ausín B., Canuto A., Crawford M.J. (2019). Affective disorders in the elderly in different European countries: Results from the MentDis_ICF65+ study. PLoS ONE.

[5] Kraemer H.C., Noda A., O’Hara R. (2004). Categorical versus dimensional approaches to diagnosis: Methodological challenges. J. Psychiatr. Res..

[6] Haslam N. (2003). Categorical versus dimensional models of mental disorder: The taxometric evidence. Aust. N. Z. J. Psychiatry.

[7] Beard C., Millner A.J., Forgeard M.J.C., Fried E.I., Hsu K.J., Treadway M.T., Leonard C.V., Kertz S.J., Björgvinsson T. (2016). Network analysis of depression and anxiety symptom relationships in a psychiatric sample. Psychol. Med..

[8] Borsboom D. (2017). A network theory of mental disorders. World Psychiatry.

[9] American Psychiatric Association (2013). DSM-5 Diagnostic Classification. Diagnostic and Statistical Manual of Mental Disorders.

[10] Maj M. (2018). Why the clinical utility of diagnostic categories in psychiatry is intrinsically limited and how we can use new approaches to complement them. World Psychiatry.

[11] Borsboom D. (2008). Psychometric perspectives on diagnostic systems. J. Clin. Psychol..

[12] Fried E.I., Epskamp S., Nesse R.M., Tuerlinckx F., Borsboom D. (2016). What are “good” depression symptoms? Comparing the centrality of DSM and non-DSM symptoms of depression in a network analysis. J. Affect. Disord..

[13] Borsboom D., Cramer A.O.J., Schmittmann V.D., Epskamp S., Waldorp L.J. (2011). The small world of psychopathology. PLoS ONE.

[14] Levinson C.A., Zerwas S., Calebs B., Forbush K., Kordy H., Watson H., Hofmeier S., Levine M., Crosby R.D., Peat C. (2017). The core symptoms of bulimia nervosa, anxiety, and depression: A network analysis. J. Abnorm. Psychol..

[15] Mullarkey M.C., Marchetti I., Beevers C.G. (2019). Using Network Analysis to Identify Central Symptoms of Adolescent Depression. J. Clin. Child Adolesc. Psychol..

[16] Park S.-C., Kim D. (2020). The centrality of depression and anxiety symptoms in major depressive disorder determined using a network analysis. J. Affect. Disord..

[17] Borsboom D., Cramer A.O.J. (2013). Network analysis: An integrative approach to the structure of psychopathology. Annu. Rev. Clin. Psychol..

[18] McNally R.J. (2016). Can network analysis transform psychopathology?. Behav. Res. Ther..

[19] Addis M.E. (2008). Gender and depression in men. Clin. Psychol. Sci. Pract..

[20] Rodgers S., Holtforth M.G., Müller M., Hengartner M.P., Rössler W., Ajdacic-Gross V. (2014). Symptom-based subtypes of depression and their psychosocial correlates: A person-centered approach focusing on the influence of sex. J. Affect. Disord..

[21] Price E.C., Gregg J.J., Smith M.D., Fiske A. (2018). Masculine traits and depressive symptoms in older and younger men and women. Am. J. Mens Health.

[22] Murri M.B., Amore M., Respino M., Alexopoulos G.S. (2020). The symptom network structure of depressive symptoms in late-life: Results from a European population study. Mol. Psychiatry.

[23] Prince M.J., Beekman A.T.F., Deeg D.J.H., Fuhrur R., Jonker C., Kivela S.L., Lawlor B.A., Lobo A., Magnusson H., Meller I. (1999). Depression symptoms in late life assessed using the EURO-D scale: The effects of age, gender and marital status in 14 European centres. Br. J. Psychiatry.

[24] Andreas S., Härter M., Volkert J., Hausberg M., Sehner S., Wegscheider K., Rabung S., Ausín B., Canuto A., Da Ronch C. (2013). The MentDis_ICF65+ study protocol: Prevalence, 1-year incidence and symptom severity of mental disorders in the elderly and their relationship to impairment, functioning (ICF) and service utilisation. BMC Psychiatry.

[25] Mf F., Folstein S., McHugh P. (1975). “Mini-mental state.” A practical method for grading the cognitive state of patients for the clinician. J. Psychiatr. Res..

[26] Wittchen H., Strehle J., Gerschler A., Volkert J., Dehoust M.C., Sehner S., Wegscheider K., Ausìn B., Canuto A., Crawford M. (2015). Measuring symptoms and diagnosing mental disorders in the elderly community: The test–retest reliability of the CIDI65+. Int. J. Methods Psychiatr. Res..

[27] Del Barrio V., The American Psychiatric Association’s Task Force, DSM-IV (1994). Diagnostic and Statistical Manual of Mental Disorders: DSM-IV.

[28] van Borkulo C.D., Epskamp S., Robitzsch A. (2014). IsingFit: Fitting Ising models using the eLasso method. R Packag. Version 0.2.0.

[29] Epskamp S., Cramer A.O.J., Waldorp L.J., Schmittmann V.D., Borsboom D. (2012). qgraph: Network visualizations of relationships in psychometric data. J. Stat. Softw..

[30] Epskamp S., Borsboom D., Fried E.I. (2018). Estimating psychological networks and their accuracy: A tutorial paper. Behav. Res. Methods.

[31] R Core Team (2013). R: A Language and Environment for Statistical Computing.

[32] Cramer A.O.J., van Borkulo C.D., Giltay E.J., van der Maas H.L.J., Kendler K.S., Scheffer M., Borsboom D. (2016). Major Depression as a Complex Dynamic System. PLoS ONE.

[33] Madhoo M., Levine S.Z. (2016). Network analysis of the Quick Inventory of Depressive Symptomatology: Reanalysis of the STAR*D clinical trial. Eur. Neuropsychopharmacol..

[34] Contreras A., Nieto I., Valiente C., Espinosa R., Vazquez C. (2019). The Study of Psychopathology from the Network Analysis Perspective: A Systematic Review. Psychother. Psychosom..

[35] Boschloo L., van Borkulo C.D., Borsboom D., Schoevers R.A. (2016). A Prospective Study on How Symptoms in a Network Predict the Onset of Depression. Psychother. Psychosom..

[36] McWilliams L.A., Sarty G., Kowal J., Wilson K.G. (2017). A Network Analysis of Depressive Symptoms in Individuals Seeking Treatment for Chronic Pain. Clin. J. Pain.

[37] van Borkulo C., Boschloo L., Borsboom D., Penninx B.W.J.H., Waldorp L.J., Schoevers R.A. (2015). Association of Symptom Network Structure With the Course of [corrected] Depression. JAMA Psychiatry.

[38] Nadorff M.R., Drapeau C.W., Pigeon W.R. (2018). Psychiatric Illness and Sleep in Older Adults: Comorbidity and Opportunities for Intervention. Sleep Med. Clin..

[39] Suzuki K., Miyamoto M., Hirata K. (2017). Sleep disorders in the elderly: Diagnosis and management. J. Gen. Fam. Med..

[40] Schechtman K.B., Kutner N.G., Wallace R.B., Buchner D.M., Ory M.G. (1997). Gender, self-reported depressive symptoms, and sleep disturbance among older community-dwelling persons. J. Psychosom. Res..

[41] Sun Y., Shi L., Bao Y., Sun Y., Shi J., Lu L. (2018). The bidirectional relationship between sleep duration and depression in community-dwelling middle-aged and elderly individuals: Evidence from a longitudinal study. Sleep Med..

[42] Forbes M.K., Tackett J.L., Markon K.E., Krueger R.F. (2016). Beyond comorbidity: Toward a dimensional and hierarchical approach to understanding psychopathology across the life span. Dev. Psychopathol..

